# Ossifying fibromyxoid tumor of the oral cavity: rare case report and long-term follow-up

**DOI:** 10.4322/acr.2020.216

**Published:** 2020-12-08

**Authors:** Maria Eduarda Pérez-de-Oliveira, Thayná Melo de Lima Morais, Márcio Ajudarte Lopes, Oslei Paes de Almeida, Willie F. P. van Heerden, Pablo Agustin Vargas

**Affiliations:** 1 Universidade Estadual de Campinas (UNICAMP), Faculdade de Odontologia de Piracicaba, Departamento de Diagnóstico Oral, Área de Semiologia e Patologia Oral, Piracicaba, SP, Brasil; 2 University of Pretoria, School of Dentistry, Department of Oral Pathology and Oral Biology, Pretoria, South Africa

**Keywords:** head and neck neoplasms, soft tissue neoplasms, mouth

## Abstract

Ossifying fibromyxoid tumor (OFMT) is a rare mesenchymal soft tissue benign neoplasm with an uncertain line of differentiation, which arises most frequently in extremities. The head and neck region involvement is uncommon, with only ten intraoral cases published in the English-language literature. One additional case of OFMT is reported here, including a literature review of intraoral reported cases. A 45-year-old female patient presented a painless nodule involving the buccal mucosa of approximately two years duration, measuring nearly 1.3 cm in maximum diameter. The main histopathological features include ovoid to round cells embedded in a fibromyxoid matrix with a perpheral shell of lamellar bone. Immunohistochemically, the tumor showed immunoreactivity for vimentin and S100. No recurrence has been detected after 7 years of follow-up.

## INTRODUCTION

Ossifying fibromyxoid tumor (OFMT) is a rare mesenchymal soft tissue benign neoplasm with an uncertain line of differentiation and intermediate biologic behavior, which arises most frequently within the subcutaneous tissue of extremities, followed by the trunk. The head and neck region is less frequently involved, comprising about 10 to 15% of OFMTs. The tumor occurs more commonly in men than women, presenting as a small, asymptomatic mass with slow growth, mainly in middle-aged adults.^1^ ^-^ ^3^ Intraoral presentation is very rare, with only ten cases reported in the English-language literature.

OFMT was first reported by Enzinger et al.^4^ in 1989 and is characterized by bland ovoid to round cells embedded in a fibromyxoid, chondroid, or hyaline matrix with a peripheral shell of lamellar bone. It has been recognized that a small subset of OFMTs exhibit atypical features such as high cellularity and mitotic activity with aggressive clinical behavior. These have been considered as malignant OFMT.^5^ Additionally, OFMTs have morphologic and immunohistochemical features that overlap with several soft tissue tumors, making recognition of this entity relevant. This report aims to describe the histopathological and immunohistochemical features of a rare case of OFMT involving the buccal mucosa with a review of the English-language literature to characterize this lesion better.

## CASE REPORT

**Figure 1:**
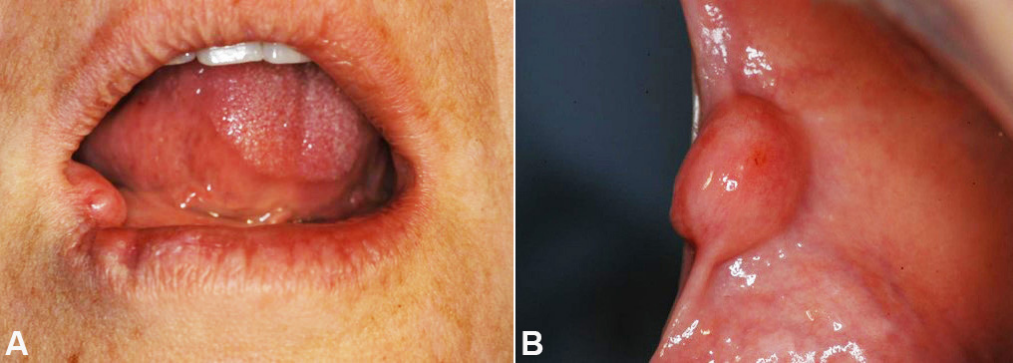
Clinical features of the intraoral OFMT. **A** – A painless nodule in the anterior region of buccal mucosa with approximately 2 years of duration; **B** – A well-circumscribed submucosal nodule with a smooth surface and normochromic in the anterior region of buccal mucosa measuring 1.3 cm in maximum diameter.

A 45-year-old female patient presented with a painless nodule in the anterior region of buccal mucosa of approximately two years duration. Intraoral examination revealed a submucosal, smooth-surfaced, well-circumscribed, and normochromic nodule, which measured nearly 1.3 cm in maximum diameter (Figure 1).

The patient’s medical history was unremarkable. The differential clinical diagnoses were fibrous hyperplasia and pleomorphic adenoma. Based on these hypotheses, the lesion was excised, and the surgical specimen sent for histopathological analysis.

**Figure 2:**
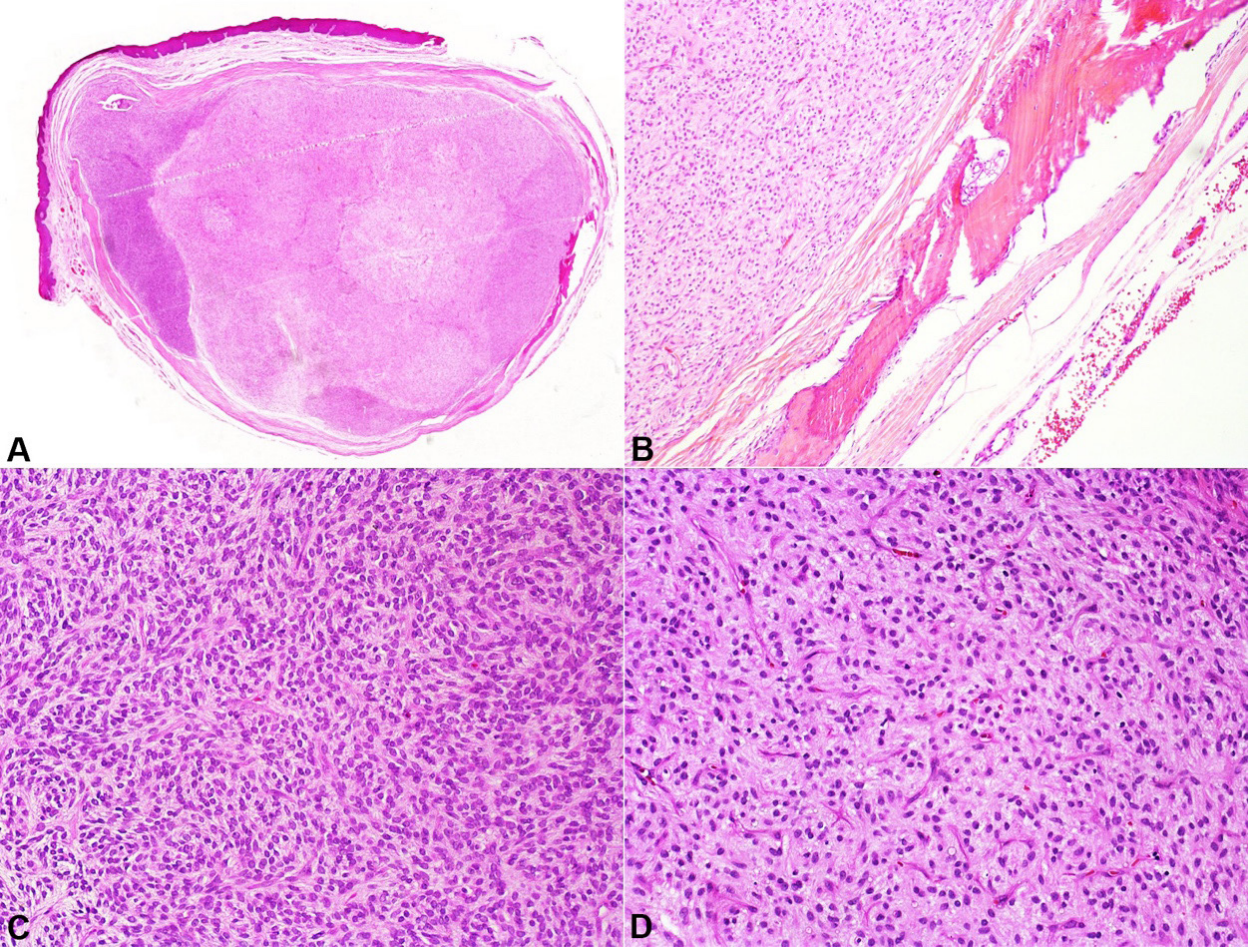
Photomicrographs of the intraoral ossifying fibromyxoid tumor. **A** – A well-circumscribed tumor with a complete fibrous capsule that extended fibrous septa, separating the hypercellular area from other areas (H&E, 1.44x); **B** – Peripheral shell of lamellar bone within the fibrous capsule (H&E, 5x); **C** – The cells range from small round to oval to fusiform shapes, with bland nuclei with fine chromatin and indistinct cytoplasm borders. The hypercellular area exhibited less intercellular matrix, often showing nuclear overlapping (H&E, 20x); **D** – Intermediate cellularity (H&E, 20x).

**Figure 3:**
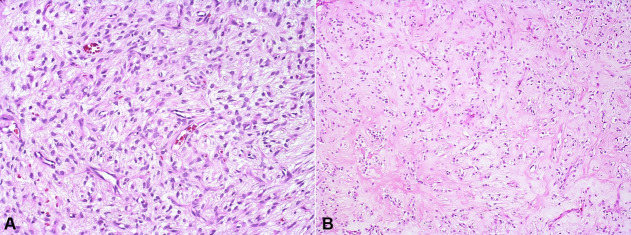
Photomicrographs of the intraoral ossifying fibromyxoid tumor. **A** – Hypocellular area with abundant fibromyxoid matrix (H&E, 20x); **B** – Hyaline material in the center of the lesion resembling chondroid or osteoid material (H&E, 10x).

Microscopically, hematoxylin-eosin stain sections showed a well-circumscribed tumor with a complete fibrous capsule that extended fibrous septae into the tumor, separating the hypercellular areas from moderate and hypocellular areas. Bone deposition, with hematopoietic marrow formation, was observed within the fibrous capsule at the periphery of the tumor. Tumor cellularity varied from low and moderate to high. The cells were arranged in sheets, ranging from small round to oval to fusiform in shape, containing bland oval to round nuclei with fine chromatin and indistinct cytoplasmic borders. The hypercellular areas showed nuclear overlapping and less intercellular matrix, while the hypocellular areas presented abundant fibromyxoid stroma. Hyaline areas, resembling chondroid or osteoid material, were found in the center of the lesion. Additionally, the stroma presented fine vessels, mainly in the myxoid and hyaline areas. Few mitoses were also found, particularly in hypercellular areas (Figure 2 and Figure 3).

**Figure 4:**
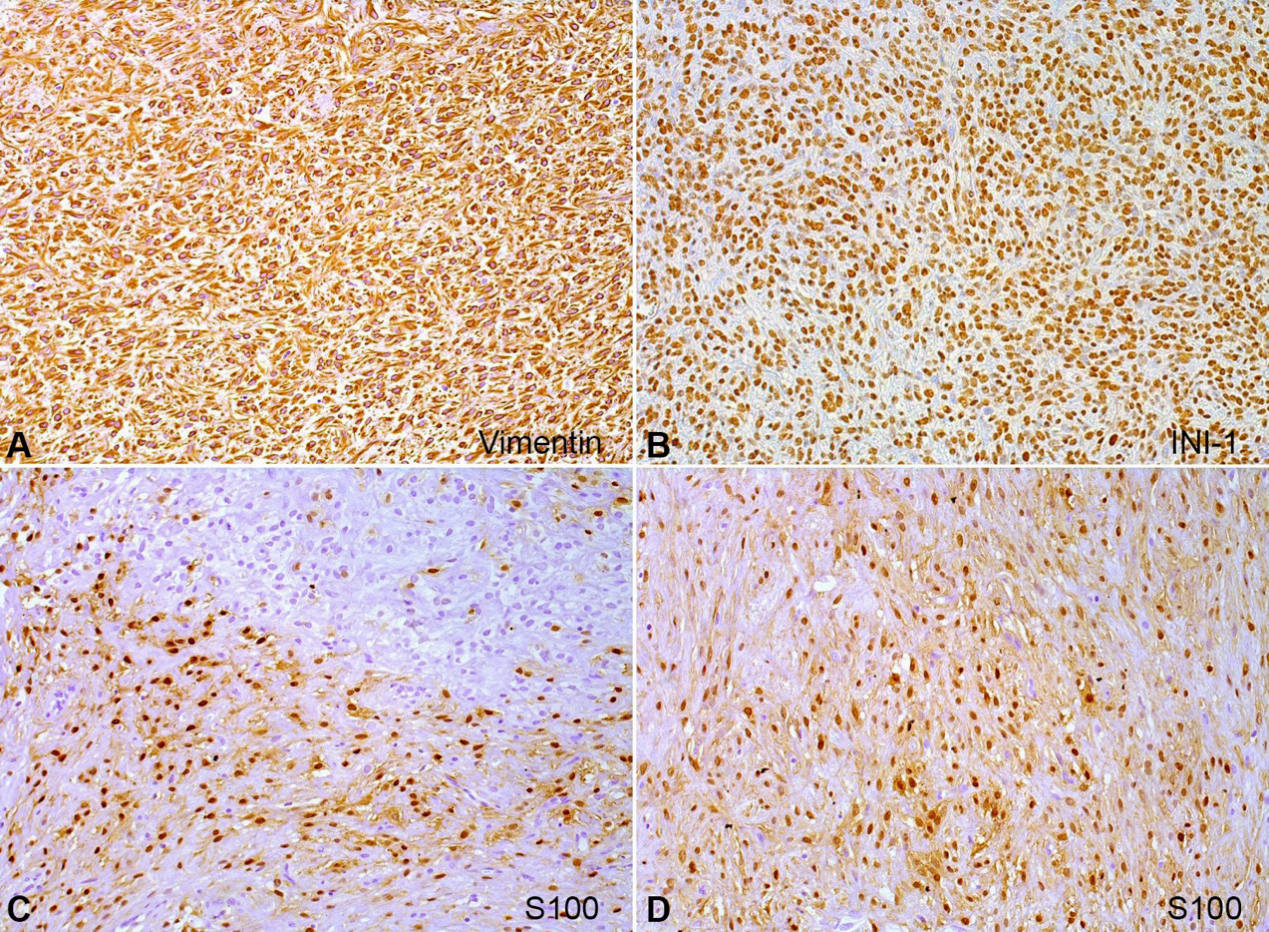
Immunohistochemical reactions of the intraoral ossifying fibromyxoid tumor. **A** – Vimentin showed cytoplasmic positivity in all tumor cells (20x); **B** – Intact nuclear expression of INI-1 in all tumor cells (20x); **C** – S100 exhibit nuclear positivity in the tumor cells; **D** – mainly in fibromyxoid and hyaline areas (20x).

**Figure 5:**
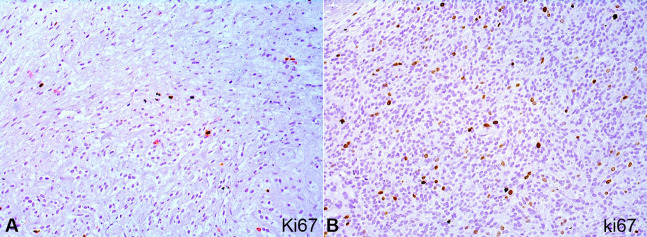
Immunohistochemical reactions of the intraoral ossifying fibromyxoid tumor. **A** – Ki67 was low (< 5%); **B** – however, in the hypercellular area had an increased rate (< 10%) (immunohistochemistry, 20x).

The tumor cells showed immunoreactivity for vimentin and S100 protein, and negativity for pan-cytokeratin (AE1/AE3), GFAP, CD34, calponin, and P63. INI-1 nuclear expression was conserved in all tumor cells (Table 1, Figure 4, and Figure 5). Based on the histopathological and immunohistochemical features, a final diagnosis of intraoral OFMT was made. No recurrence has been detected after 7 years of follow-up.

**Table 1:** Immunohistochemical profile of the present intraoral ossifying fibromyxoid tumor

**IHC stain**	**Result**	**Pattern**
AE1/AE3	Negative	NA
GFAP	Negative	NA
S100	Focally positive	Nuclear, mainly in hypocellular and hyaline areas
Vimentin	Positive	Cytoplasmic, all tumor cells
Calponin	Negative	NA
P63	Negative	NA
CD34	Negative	NA
INI-1	Conserved	Nuclear, all tumor cells
Ki-67	Low	Nuclear, < 5% of tumor cells in hypocellular and moderate areas, and < 10% in hypercellular area.

IHC stain – immunohistochemical stain; NA – not applicable.

## DISCUSSION

OFMT is a rare neoplasm of soft tissues that arises more commonly in the extremities and trunk. The head and neck region is involved in only 10 to 15% of the cases. Clinically, most OFMT presents as a small, nodular, painless lesion with slow growth.^1^ ^,^ ^6^ A comprehensive review of the English-language literature identified only ten published cases of intraoral OFMT (Table 2). The real incidence of this lesion in the mouth may be underestimated, since proper diagnosis is difficult, considering the clinical, microscopical, and immunohistochemical features overlapping with several other lesions.

**Table 2:** Cases of intraoral ossifying fibromyxoid tumor reported in the English-language literature

**Author**	**Age y/ Sex**	**Location**	**Clinical appearance**	**Size (cm)**	**Time of complaint**	**Ossification**	**Mitoses**	**Atypia**	**Immunohistochemistry**	**INI-1**	**Follow-up; Status**
Schofield et al. ^7^	39/M	Lip	ovoid masses	1.5	slow growing	NA (ITS: present in 12/13 cases)	0-1/10 HPFs	No	ITS: S100 10/12, Desmin 7/10, SMA 4/8, fast myosin and sarcomere actin: 1/6, cytokeratin 1/7, vimentin 3/3, GFAP 1/3, Leu-7 1/3, collagen IV 2/3, HMFG2 1/3, MSA−, PGP 9.5−, factor VIII−, CD34−, HMW, keratin−.	NA	NA (ITS: Follow-up were available on 8/13 (1-10 years – median: 7 years); NA (ITS: NED)
	41/M	Buccal mucosa		1.5							
Williams et al.^8^	67/F	Left mandibular vestibule	NA	1.0	4 months	NA (in their series: present in 6/8 cases)	1-2/10 HPFs	No	ITS: S100 (3/5), NSE (3/5), GFAP (2/5), Vimentin (5/5), Leu-7 3/5, SMA (2/5), MSA (2/5), NFP (0/5), EMA (0/5), Cytokeratin (0/5).	NA	1.5 years; NED
	37/F	Soft palate		4.5	NA						3 years; NED
Mollaoglu et al.^9^	13/M	Left mandibular vestibule	Hard, warm, painless mass covered by normal mucosa	2.0	4 months	Yes	No	No	Vimentin +, SMA+ (focally), GFAP + (focally), S100−, EMA−, Pankeratin−, Desmin−, Leu-7−, CD34−.	NA	NA; NED
Miettinen et al.^1^	NA/NA	Lower lip	NA	NA	NA	NA	NA	NA	ITS: Vimentin (33/33), S100 (67/71), CD10 (22/28), Keratin (6/45), Collagen IV (3/23), Desmin 4/40, GFAP (3/41), EMA (1/47), SMA (1/43), CD34 (0/38), HMB45 (0/13)	NA	NA
Sharif et al.^10^	14/F	Between buccal and gingival mucosa in the left anterior mandibular region	Nodular swelling with reddish surface.	4.0	3 months	Yes	<2/10 HPFs	No	Vimentin +, S100 +, EMA −, cytokeratins −	NA	NA
Nonaka et al.^11^	21/F	Posterior mandibular gingiva	Painless exophytic mass with a reddish and lobulated surface.	6.0	6 months	Yes	No	No	Vimentin +, S100 +, SMA −, MAS −, GFAP −	NA	7 months; NED
Ohtaet al.^12^	26/M	Dorsal tongue	1^st^ lesion: painless nodule 2^nd^ lesion: painless mass with reddish and lobulated surface.	1^st^ lesion: 0.7; 2^nd^ lesion: 2.0	1^st^ lesion: 2 weeks; 2^nd^ lesion: NA - gradually increasing	Yes	1^st^ lesion: low; 2^nd^ lesion: >2/10 HPFs	1^st^ lesion: No; 2^nd^ lesion: High cellularity, scant pleomorphism	Vimentin+, S100+ (partially), GFAP−, cytokeratins−, αSMA−, calponin−, desmin−, CD68−, CD34−, p63−, Ki67 (7%)		1^st^ lesion: 48 months; Recurrence (malignant OFMT – no metastasis) 2^nd^ lesion: NA
Titsinideset al.^13^	13/M	Retromolar trigone area	Painless mass, hard, nonmoveable covered by normal mucosa.	0.8	7 months	Yes	No	No	Vimentin +, NSE+, MSA+, S100 −, GFAP −, SMA−, desmin −, AE1/AE3−, CD99−, CD34−	NA	48 months; NED
index case	45/F	Buccal mucosa	Painless nodule with smooth- surface.	1.3	24 months	Yes	<2/10 HPFs	Mild pleomorphism	Vimentin +, S100 +, GFAP −, CD34 −, AE1/AE3 −, Calponin −, P63 −, Ki-67 5%	Conserved	7 years; NED

EMA – Epithelial membrane antigen; F – Female; GFAP – Glial fibrillary acidic protein; HPF – High-power field; INI-1 – gene INI-1; ITS - In their series; M – Male; MSA – Muscle specific actin; NA – Not available; NFP – Neurofilament protein; NED – No evidence of disease; NSE – Neuron specific enolase; SMA – Smooth muscle-actin; y – year.

A literature review on oral OFMT, including the current case, revealed a mean age of 31.6 years (ranging from 13^9^ ^,^ ^13^ to 67^8^ years), with a male: female ratio of 1.5:1. The most common clinical presentation was a painless nodule covered by normal mucosa and smooth surface. Three cases were described with a reddish surface.^10^ ^-^ ^12^ The tumor presented as slow-growing, with a mean time of 6.9 months before diagnosis, except for one case that had a history of two weeks. This case was diagnosed as a malignant OFMT.^12^ The mean size of the lesions was 2.3 cm, ranging from 0.8 cm^13^ to 6.0 cm.^11^ These data were similar to extraoral cases of OFMT.^1^ ^-^ ^3^ Soft tissues of the mandible were the most commonly involved sites, including lower vestibule (3 cases), posterior lower gingiva (1 case), and retromolar trigone (1 case). However, it seems that any site of the oral mucosa can be affected, such as lip (2 cases), buccal mucosa (2 cases), soft palate (1 case), and dorsal tongue (1 case). Clinically, intraoral OFMT has several clinical differential diagnoses, including reactive and neoplastic lesions. Reactive lesions may include fibrous hyperplasia and deep mucocele. Among neoplastic lesions, mesenchymal and salivary gland tumors usually are considered in clinical differential diagnoses, such as lipoma^14^ and pleomorphic adenoma.^15^

OFMT is usually a well-circumscribed lesion with a partial or complete fibrous capsule that can produce fibrous septa. The main histopathological feature is the presence of a peripheral shell of lamellar bone within the fibrous capsule. Rarely, this bone may contain hematopoietic marrow,^1^ ^,^ ^3^ as observed in the current case. Some atypical OFMT cases may not present mature bone and osteoid,^5^ although all intraoral OFMT cases reported the presence of bone formation. In high cellularity areas, mild to severe atypia and mitoses can be found.^1^ Five OFMT cases of the oral cavity reported mitotic activity, and in one case, it was considered high in the recurrent lesion.^12^ Folpe and Weiss^16^ in 2003, proposed a risk stratification system for OFMT in which cases with a high nuclear grade, high cellularity and mitotic activity greater than 2 mitotic figures/50 HPF should be classified as “malignant OFMT”. Cases with atypical features, but not with all the criteria listed above, may be classified as “atypical OFMT” and considering the others as “typical OFMT”.^2^ Despite the presence of focal hypercellular areas, the mitotic activity was minimal. Hence, the current case was considered as a typical OFMT. Most of the reported intraoral cases were typical OFMTs, with only one exception, in which the recurrent tumor was classified as malignant OFMT.^12^

The immunohistochemical (IHC) profile of intraoral OFMT is similar to cases arising in extraoral sites,^3^ with frequent positivity for vimentin (6/6), and S100 (4/6). Vimentin has been reported as being diffusely positive, while S100 expression was only focal, similar to the present case. GFAP and SMA showed focal immunoreactivity in one case each, but not in the present case. Other markers that can occasionally be expressed in extraoral OFMT cases (usually focally) are neurofilament, neuron-specific enolase (NSE), CD56, CD10, and MSA. Single case positivity for MSA and NSE was reported in intraoral OFMT cases. Cytokeratins, AE1/AE3, desmin, CD34, calponin, p63, EMA, CD68, and CD99 were negative. Epithelial markers (cytokeratins, pan-cytokeratins, and EMA) have been described with focal and weak expression in cases with reduced expression of S100, particularly in malignant OFMT cases,^2^ ^,^ ^3^ however, a single case report of malignant OFMT in the oral cavity showed focal positivity for S100 and negativity for cytokeratins.^12^ The IHC profile was supportive of neuroectodermal differentiation of this lesion, although myoepithelial differentiation has also been suggested.^2^ ^,^ ^6^ However, in the broad profile of IHC of this lesion, it was difficult to determine the precise line of differentiation.

The range of differential histological diagnosis is broad, particularly in atypical cases of OFMT. It includes myxoid neurofibroma, peripheral nerve sheath tumor, low-grade fibromyxoid sarcoma, solitary fibrous tumor, ectomesenchymal chondromyxoid tumor (ECT), myoepitheliomal tumors, and glomus tumor (GT). Myoepithelioma is a salivary gland tumor formed by neoplastic myoepithelial cells that exhibit different cell shapes that varies from round to ovoid to fusiform surrounded by fibrous, hyaline, or fibromyxoid matrix. However, no chondroid or osseous metaplasia are found and myoepithelial markers, such as P63 and calponin, are positive.^17^ ECT also displays round to ovoid to fusiform cells with chondroid formation surrounded by fibromyxoid matrix, differing from OFMT in multilobulated aspect and absence of fibrous capsule and osteoid or bone formation.^18^ Furthermore, ECT affects almost exclusively the tongue and is positive for GFAP.^18^ ^,^ ^19^ GT is characterized by an encapsulated proliferation of epithelioid cells surrounded by numerous vessels with different sizes in a myxoid stroma. The neoplastic cells of GT are negative for S100.^20^ Additionally, all tumors mentioned above are not associated with the presence of a peripheral layer of lamellar bone.

Several studies have identified by IHC or fluorescence in situ hybridization (FISH) a mosaic pattern loss of INI-1 expression (integrase interactor 1/ SMARCB1/ hSNF5/ BAF47) in approximately 30 to 60% of neoplastic cells in up to three-quarters of extraoral OFMT cases.^2^ ^,^ ^21^ INI-1 is a tumor suppressor gene located on chromosome 22q11.2 that encodes a protein expressed essentially in all nucleated cells.^22^ The mosaic loss inactivation of this tumor suppressor gene in OFMT, including typical and malignant forms, has also been suggested as a role in tumorigenesis.^2^ However, INI-1 conserved expression has been identified in other studies, and the molecular mechanisms of this event in OFMT remain unclear.^23^ To the best of our knowledge, this is the first intraoral OFMT case reported in the English-literature that analyzed the immunoreactivity of INI-1. An intact nuclear expression was observed in all tumor cells.

OFMTs have been characterized as tumors with intermediate clinical behavior, although the majority behave in a benign manner. Some tumors present with local recurrence or rarely metastasizing to distant locations. Surgical excision is reported as the treatment of choice for OFMT.^1^ ^,^ ^3^ The majority of reported intraoral cases followed a benign course, with no evidence of recurrence or metastasis. Miettinen et al.^1^ reported that 22% of 104 cases had recurred, which usually manifested more than 10 years after the initial diagnosis, emphasizing that OFMT has a potential for late local recurrence, but no metastases were detectable in these cases. The locally recurrent tumors have been described with similar features as the nonrecurrent lesions, although some recurrent cases can exhibit increased cellularity and mitotic activity.^24^ An intraoral OFMT case reported by Ohta et al.^12^ recurred after 4 years of the primary diagnosis, presenting higher cellularity and mitotic activity, resulting in the recurrent tumor being diagnosed as a malignant OFMT. In a study conducted by Graham et al.,^2^ 33% of patients diagnosed as malignant OFMT had adverse events, in which 2 patients had local recurrence, 3 had distant metastases, and 3 died from the disease. Finally, one intraoral case was reported as malignant OFMT, without evidence of metastatic tumor.^12^

## CONCLUSION

In summary, OFMT is a soft tissue benign mesenchymal neoplasm with intermediate biologic behavior and rarely involves the oral cavity. Despite its usual benign clinical course, the correct diagnosis is important due to the risk of late local recurrence and eventual metastasis, although the latter not yet reported in intraoral cases. Thus, it is important to consider this lesion in both clinical and histopathological diagnosis of fibromyxoid soft neoplasms in the oral cavity and to have a long-term follow-up.
